# Ultrasound-Assisted Nanoemulsification for Food and Nutraceutical Applications: Mechanisms, Formulation Strategies, and Translational Challenges

**DOI:** 10.3390/foods15193456

**Published:** 2026-09-27

**Authors:** Xingyou Mo, Qian Li, Xiaoyan Sun, Abdul Rahaman, Ankita Kumari

**Affiliations:** 1 School of Food & Pharmaceutical Science and Technology, Guangzhou College of Technology and Business, Guangzhou 510850, China; 2 School of Food Science and Engineering, Foshan University, Foshan 528225, China; 3 Guangdong Key Laboratory of Food Intelligent Manufacturing, Foshan University, Foshan 528225, China; 4 School of Food Science and Engineering, South China University of Technology, Guangzhou 510641, China

**Keywords:** ultrasound, acoustic cavitation, nanoemulsions, essential-oil nanoemulsions, bioactive delivery systems, antimicrobial food preservation

## Abstract

Ultrasound-assisted nanoemulsification has emerged as a versatile platform for producing food-grade nanoemulsions with enhanced stability and improved delivery potential of lipophilic bioactives. This review offers a critical, integrated analysis of the mechanistic principles, formulation parameters, applications, and translational challenges of ultrasound-assisted nanoemulsification for food and nutraceutical applications. The review synthesizes findings on cavitation-driven droplet disruption, key operational parameters, and formulation factors governing nanoemulsion formation and performance. It also discusses applications in bioactive delivery, essential-oil-based preservation, and protein-based carrier systems. Key findings reveal that ultrasound can effectively produce sub-200 nm droplets through mechanical cavitation-driven disruption. However, excessive processing provides limited size reduction, whereas formulation composition determines emulsion stability and functionality. The review identifies that improved in vitro bioaccessibility does not constitute evidence of enhanced systemic bioavailability. Droplet size is a conditional rather than universal predictor of gastrointestinal delivery, and the interface paradox represents a fundamental design challenge that requires rational interfacial engineering. Persistent barriers to industrial translation include inconsistent parameter reporting, non-uniform cavitation fields during scale-up, and limited validation in complex food matrices. Future research priorities include standardized reporting of acoustic parameters, in vivo validation linking bioaccessibility with systemic bioavailability, pilot-scale studies, and interfacial engineering strategies to resolve the stability–digestibility trade-off.

## 1. Introduction

Ultrasound-assisted nanoemulsification is gaining attention as a promising non-thermal approach for producing food-grade nanoemulsions with better physical stability and improved ability to deliver lipophilic bioactive compounds. Nanoemulsions are kinetically stable colloidal systems formed from two immiscible liquid phases, in which the dispersed droplets are usually within the range of about 20–200 nm. Because of their nanoscale structure, they offer several advantages over conventional emulsions, including a larger interfacial area, greater resistance to gravitational separation, adjustable optical and rheological properties, and better dispersion of poorly water-soluble compounds in aqueous food systems [1,2,3]. These features make nanoemulsions useful delivery systems for essential oils, carotenoids, polyphenols, fat-soluble vitamins, and polyunsaturated fatty acids, with potential applications in functional foods, nutraceuticals, beverages, edible coatings, and active packaging [1,3]. At the same time, growing consumer demand for minimally processed and clean-label foods has increased interest in advanced emulsification methods that can enhance ingredient performance without compromising product quality [3,4].

Producing food-grade nanoemulsions is still technically demanding because immiscible liquids, such as oil and water, resist being broken into very small droplets due to strong interfacial forces. Low-energy methods, such as phase inversion and spontaneous emulsification, can form small droplets when the formulation conditions are carefully controlled. However, these methods often depend on very specific compositions and may require relatively high levels of emulsifier, which can limit their use in some food products [3,4]. High-energy methods, including high-pressure homogenization, microfluidization, and ultrasonication, address this challenge by applying mechanical forces that break larger droplets into nanoscale structures [3,5]. Among these techniques, high-intensity ultrasound has attracted particular interest in food engineering because it can efficiently disrupt interfaces, uses relatively simple equipment, is suitable for heat-sensitive ingredients, and can be adapted for both batch and continuous processing systems [5,6].

The key mechanism behind ultrasound-assisted nanoemulsification is acoustic cavitation, where alternating pressure waves create, grow, and collapse tiny bubbles in the liquid. When these bubbles collapse, they generate intense local forces, such as shear, microjets, shock waves, and microstreaming, which break the oil–water interface into much smaller droplets and support nanoemulsion formation [6,7]. Recent work has moved this technique beyond basic process development toward food and nutraceutical applications, especially for delivering lipophilic compounds such as curcumin, resveratrol, vitamins, carotenoids, and essential oils. In these systems, processing conditions, including frequency, power, sonication time, and phase composition, strongly affect droplet size and distribution [8,9,10,11,12,13,14]. Essential-oil nanoemulsions are especially promising for antimicrobial food preservation, active packaging, and natural protection strategies, as ultrasound-assisted formulations can improve their antimicrobial performance and preservation potential [12,15,16,17,18,19,20,21]. In addition, high-intensity ultrasound has been explored for modifying protein structures and improving protein-based interfacial functionality, creating opportunities for novel food-grade nanoemulsion systems [22]. However, despite substantial progress, several challenges continue to limit industrial translation, including acoustic-field heterogeneity during scale-up [23], the interface paradox whereby cavitation-induced oxidative reactions may compromise product quality [7,24], and the limited evidence linking improved in vitro bioaccessibility with systemic bioavailability [3,25]. These developments highlight the versatility of ultrasound as an interfacial engineering platform for designing functional nanoemulsions with tailored physicochemical and biological properties.

Despite these advances, wider use of ultrasound-assisted nanoemulsification in food systems still faces important challenges. Although acoustic cavitation is known to drive droplet breakup, the links between cavitation behaviour, interfacial restructuring, and final nanoemulsion performance are still not fully understood, especially in complex food matrices containing proteins, polysaccharides, polyphenols, or particle-based stabilizers [26,27]. Sonochemical effects also need careful attention. Reactive oxygen species generated during cavitation may alter interfacial properties, but excessive formation can accelerate lipid oxidation and degrade oxidation-sensitive bioactive compounds [27,28]. Therefore, smaller droplets do not automatically mean better functionality; optimization must balance physical stability with chemical protection.

Several reviews have discussed ultrasound-assisted emulsification, including process parameters and droplet formation [3,29], ultrasonic devices and scale-up [23], essential-oil nanoemulsions [21,30] and protein modification [22,31]. However, fewer reviews bring these areas together by linking acoustic cavitation mechanisms with formulation-dependent performance, functional validation, oxidative stability, and industrial translation. In particular, the connections among interfacial structure, digestion behaviour, oxidative sensitivity, and the difference between bioaccessibility and bioavailability still need more critical evaluation.

Therefore, this review critically examines ultrasound-assisted nanoemulsification for food and nutraceutical applications. It explains the mechanisms behind cavitation-driven droplet breakup and interfacial restructuring, evaluates how processing and formulation factors affect nanoemulsion formation, stability, and functionality, and discusses recent applications in bioactive delivery, essential-oil-based preservation, active packaging, and protein-based systems. It also highlights major challenges, including parameter standardization, oxidative stability, scale-up, energy efficiency, and regulatory issues. By connecting cavitation science with formulation design and industrial needs, this review aims to support the practical development of ultrasound-assisted nanoemulsions for food applications.

## 2. Acoustic Cavitation and the Sonochemical Mechanism of Nanoemulsification

### 2.1. Cavitation-Driven Droplet Disruption and Interfacial Restructuring

Ultrasound-assisted nanoemulsification is primarily governed by acoustic cavitation, which occurs when high-intensity sound waves generate alternating compression and rarefaction cycles within a liquid. These pressure fluctuations promote the formation, growth, and collapse of cavitation bubbles originating from dissolved gases or pre-existing nuclei. During inertial collapse, cavitation generates localized mechanical effects, including intense shear forces, shock waves, microjets, and turbulent microstreaming, which collectively provide the energy required for interfacial disruption and droplet refinement [7,32]. Although extreme temperature and pressure conditions have been estimated within collapsing bubbles, these effects are highly localized and represent transient energy-concentration events rather than bulk processing conditions.

In oil-in-water emulsification, ultrasound-induced droplet disruption occurs through sequential mechanical processes. Initially, acoustic streaming and turbulence generated around the ultrasonic source enhance mixing and reduce larger droplets. Subsequently, localized cavitation-induced stresses deform and fragment droplets when disruptive forces overcome interfacial resistance. Newly formed droplets must then be rapidly stabilized by surfactants, proteins, or other interfacial materials to prevent recoalescence and achieve kinetically stable nanoemulsions [26,33]. Therefore, nanoemulsion formation depends not only on cavitation intensity but also on the ability of the formulation to stabilize newly created interfaces. The resulting nanoemulsions remain thermodynamically unstable but exhibit improved kinetic stability due to reduced droplet size, enhanced Brownian motion, and protective interfacial layers providing steric or electrostatic stabilization [1,3]. These physicochemical characteristics determine the stability and functional behaviour of ultrasound-generated nanoemulsion systems (Figure 1).

### 2.2. Influence of Ultrasound Parameters on Nanoemulsion Formation

The efficiency of ultrasound-assisted nanoemulsification depends strongly on acoustic conditions, particularly frequency, intensity, and delivered energy. Frequency influences cavitation behaviour and determines the balance between mechanical disruption and sonochemical activity. Low-frequency ultrasound (approximately 20–40 kHz) generally produces larger cavitation bubbles with more violent collapse events, generating stronger mechanical forces suitable for droplet disruption. In contrast, higher frequencies favour more stable cavitation and increased radical formation, making them more relevant for sonochemical reactions rather than physical emulsification [34]. Consequently, low-frequency probe systems, particularly around 20 kHz, are widely employed for food nanoemulsion preparation because of their effective mechanical action [26,34].

Acoustic intensity influences cavitation strength and therefore droplet disruption efficiency. However, nominal electrical power alone does not accurately represent the energy transferred to the liquid because energy delivery depends on reactor geometry, probe characteristics, liquid composition, and operating conditions. Increasing acoustic intensity generally improves droplet disruption until an optimum condition is reached, beyond which additional energy contributes mainly to heat generation and provides limited improvement in droplet size reduction [35,36]. Consequently, accurate reporting of acoustic power, specific energy input, processing volume, and temperature conditions is essential for reproducible comparison among studies.

A persistent limitation in ultrasound-assisted nanoemulsion research is inconsistent reporting of processing parameters. Differences in probe geometry, amplitude settings, energy measurement methods, and reactor configuration frequently hinder direct comparison between studies [7,23]. Standardized reporting of acoustic parameters is therefore necessary to improve reproducibility and facilitate rational optimization of ultrasound-based nanoemulsification.

### 2.3. Balancing Mechanical and Sonochemical Effects

Although mechanical effects dominate ultrasound-assisted emulsification, cavitation also generates chemical effects through water sonolysis and the formation of reactive oxygen species (ROS), including hydroxyl radicals and hydrogen peroxide [7]. These reactive species may influence nanoemulsion properties depending on formulation composition and processing intensity. Controlled sonochemical activity can contribute to interfacial modification and mass transfer; however, excessive ROS generation may promote oxidation of susceptible lipid components and reduce the stability of oxidation-sensitive compounds, particularly in oil-based delivery systems [37,38].

This balance is particularly important for formulations containing oxidation-prone oils or bioactive compounds. Excessive sonication may improve droplet disruption while simultaneously increasing oxidative stress, whereas optimized processing conditions combined with suitable protective strategies can minimize undesirable reactions [28]. Therefore, ultrasound-assisted nanoemulsification should be considered a balance between mechanical energy delivery and chemical reactivity, requiring simultaneous optimization of acoustic conditions and formulation characteristics.

## 3. Operational and Formulation Parameters Governing Ultrasound-Assisted Nanoemulsification

### 3.1. Sonication Time and Amplitude

Sonication time is one of the most frequently investigated operational parameters in ultrasound-assisted nanoemulsification because it determines the duration of cavitation exposure and cumulative energy delivered to the emulsion system. In most formulations, droplet size decreases rapidly during the initial stages of sonication due to cavitation-induced shear forces and interfacial disruption, followed by a plateau when droplet fragmentation becomes balanced by stabilization and recoalescence processes. Ghosh et al. reported that optimized 20 kHz ultrasound treatment produced a stable *Ocimum basilicum* essential-oil nanoemulsion with a final droplet size of approximately 29 nm, while prolonged treatment resulted in limited additional improvement [39]. Similar time-dependent behaviour has been reported for curcumin-, cinnamon-oil-, essential-oil blend-, and protein-stabilized nanoemulsion systems, indicating that excessive sonication does not necessarily improve droplet refinement and may increase energy consumption without proportional benefits [11,40,41,42].

Amplitude determines the displacement of the ultrasonic probe and directly influences acoustic pressure generation and cavitation intensity. Increasing amplitude generally enhances droplet disruption by increasing shear forces, turbulence, and cavitation activity, thereby promoting the formation of smaller droplets. However, the relationship between amplitude and droplet size is highly dependent on formulation composition, reactor configuration, and processing conditions. Beyond an optimum amplitude, additional acoustic energy may primarily contribute to heat generation and energy dissipation rather than further droplet reduction [13,43]. Therefore, optimization of amplitude should focus on achieving sufficient interfacial disruption while avoiding unnecessary thermal and energetic input.

### 3.2. Energy Input and Temperature Control

Ultrasound processing efficiency is more accurately described by delivered acoustic energy than by nominal electrical power alone. The fraction of energy transferred into the liquid depends on several factors, including ultrasonic frequency, probe characteristics, reactor geometry, processing volume, and physicochemical properties of the emulsion. Therefore, parameters such as calorimetric power, specific energy input, treatment time, and processing volume provide more meaningful comparisons among different ultrasound systems than generator power alone [43,44].

Increasing energy input generally promotes droplet disruption by enhancing cavitation activity; however, the relationship between energy input and droplet size reduction is not linear. Once an optimal energy level is reached, additional energy may provide limited improvement because the system approaches a balance between droplet fragmentation and stabilization mechanisms. Excessive energy input can also increase temperature, which may alter viscosity, affect interfacial behaviour, and compromise the stability of sensitive formulations [42,45].

Temperature control is therefore an important consideration during ultrasound-assisted nanoemulsification. Heat accumulation during high-intensity sonication may influence the physical properties of emulsions and accelerate degradation reactions in temperature-sensitive components. Cooling approaches, including intermittent sonication, temperature-controlled vessels, and external cooling systems, are commonly employed to maintain consistent processing conditions and preserve nanoemulsion quality.

### 3.3. Surfactant-to-Oil Ratio and Continuous-Phase Properties

Although ultrasound provides the mechanical energy required for droplet disruption, formulation composition determines whether newly generated droplets remain stable after processing. The surfactant-to-oil ratio is therefore a critical parameter controlling interfacial coverage, droplet size, and emulsion stability. Insufficient surfactant concentration may result in incomplete coverage of newly generated interfaces, increasing the probability of droplet recoalescence, whereas excessive surfactant addition generally provides diminishing improvements once adequate interfacial stabilization has been achieved [46,47].

The physicochemical characteristics of the emulsifier also influence nanoemulsion formation. Non-ionic surfactants, such as Tween 20 and Tween 80, are widely used in food nanoemulsions because of their rapid adsorption at oil–water interfaces and ability to provide steric stabilization. In protein-based systems, ultrasound treatment may induce controlled structural modification of proteins, improving their adsorption behaviour and interfacial functionality. Studies involving soy protein isolate, whey proteins, and plant-protein-based systems have demonstrated that optimized ultrasound treatment can enhance emulsifying properties and facilitate formation of smaller droplets [27,48,49]. The properties of the continuous phase further influence cavitation behaviour and droplet disruption. Moderate increases in viscosity may improve emulsion stability by reducing droplet collision and recoalescence; however, excessive viscosity can attenuate acoustic propagation and reduce cavitation efficiency, limiting droplet break-up [50,51]. Consequently, the optimum ultrasound conditions depend on the combined effects of acoustic parameters, emulsifier characteristics, dispersed-phase composition, and continuous-phase properties (Figure 1).

## 4. Applications of Ultrasound-Assisted Nanoemulsions in Food and Nutraceutical Delivery

### 4.1. Ultrasound-Assisted Nanoemulsions: From Droplet Engineering to Bioaccessibility

The functional performance and translational challenges of ultrasound-assisted nanoemulsions are summarized in Figure 2. Ultrasound-assisted nanoemulsification is increasingly used to improve the delivery of lipophilic bioactive compounds in foods and nutraceuticals. Its value lies not only in reducing droplet size but also in shaping the oil–water interface, which strongly influences the gastrointestinal fate of encapsulated compounds. Through acoustic cavitation, ultrasound generates intense local shear, microjets, and microstreaming that break coarse emulsions into fine droplets, often below 200 nm under optimized conditions. However, droplet size and stability depend on more than ultrasound settings alone; oil composition, emulsifier type, formulation properties, and energy input are equally important [14,36]. The larger interfacial area of nanoemulsions can improve digestive enzyme access, support lipid hydrolysis, and increase the transfer of lipophilic compounds into mixed micelles, which is essential for intestinal bioaccessibility [8,9]. However, higher bioaccessibility does not automatically mean higher systemic bioavailability, because absorption, metabolism, and tissue distribution also determine how much of the compound becomes biologically available.

The same principle has also been shown for other lipophilic food bioactives. Walia et al. prepared fish-oil-based vitamin D nanoemulsions using ultrasonication and reported high encapsulation efficiency (95.7–98.2%), strong storage stability, and greater vitamin D recovery after simulated gastrointestinal digestion than non-encapsulated vitamin D [45]. Similarly, ultrasound-assisted κ-carrageenan-stabilized nanoemulsions containing fucoxanthin-rich oil showed improved bioaccessibility after simulated digestion [16]. Although curcumin, vitamin D, and fucoxanthin differ structurally, these studies suggest a common mechanism: ultrasound mainly improves delivery by reshaping the interface and supporting lipid digestion, rather than through compound-specific interactions.

These results are promising, but their translational value should be interpreted carefully. Most studies still focus on encapsulation efficiency, release behaviour, antioxidant retention, or simulated gastrointestinal bioaccessibility. Far fewer include animal pharmacokinetic studies, and human evidence for ultrasound-fabricated food nanoemulsions remains limited. Therefore, improved micellarization should be viewed as evidence of better delivery potential, not as definitive proof of higher systemic bioavailability [8,9].

Overall, current evidence supports acoustic cavitation as a useful tool for engineering nanoemulsion interfaces that improve the physical stability and gastrointestinal delivery of lipophilic food bioactives. However, performance depends strongly on formulation composition, interfacial structure, and digestive conditions. Future studies should combine standardized in vitro digestion with pharmacokinetic validation using compositionally identical sonicated and non-sonicated formulations, so the specific contribution of acoustic cavitation can be separated from formulation effects.

### 4.2. Droplet Size as an Important but Conditional Determinant of Gastrointestinal Delivery

Droplet-size reduction is one of the main structural effects of ultrasound-assisted nanoemulsification, but its role in gastrointestinal delivery is not universal. Acoustic cavitation generates local shear, microjets, and turbulent forces that break coarse emulsions into smaller droplets with a much larger oil–water interfacial area. This expanded interface can improve the adsorption of bile salts, colipase, and pancreatic lipase, thereby supporting lipid hydrolysis and the transfer of lipophilic compounds into mixed micelles, which is essential for intestinal bioaccessibility [8,9]. However, the droplet size measured immediately after ultrasonication only reflects the starting structure of the system. Its actual digestive performance depends on whether the interface remains stable, accessible, and digestible during gastric and intestinal digestion.

Several ultrasound-assisted studies illustrate this point. Xu et al. produced soybean-oil nanoemulsions using an ultrasonic microreactor, achieving a mean droplet size of about 96 nm and good storage stability for 45 days [14]. However, the study focused mainly on process optimization and colloidal stability, rather than digestion or nutrient absorption. Similarly, Abbas et al. showed that ultrasound conditions, formulation composition, and OSA-modified starch concentration strongly influenced the physicochemical properties of curcumin nanoemulsions [10]. Sharma et al. also optimized ultrasound-assisted curcumin nanoemulsions, producing droplets of about 139 nm with high encapsulation efficiency and controlled release during simulated digestion [11]. Together, these studies confirm that ultrasound can produce stable nanoscale emulsions, but they do not prove that smaller droplets alone are responsible for improved bioaccessibility, because interfacial composition and digestive behaviour were not independently controlled.

Vitamin D delivery further shows why formulation design matters as much as particle size. Ultrasonicated fish-oil-based vitamin D nanoemulsions had larger droplets, around 300–450 nm, yet still showed high encapsulation efficiency and significantly improved vitamin D bioaccessibility during simulated digestion [45]. This suggests that there is no fixed droplet-size threshold for optimal gastrointestinal delivery. Instead, delivery performance depends on several interacting factors, including carrier-oil composition, emulsifier organization, lipid digestibility, and the efficiency of mixed-micelle formation [8,9].

Evidence from non-ultrasonic nanoemulsions supports the same idea. Docosahexaenoic acid (DHA)-rich nanoemulsions produced by microfluidization with similar initial droplet sizes, approximately 131–142 nm, showed different lipolysis behaviour and DHA bioaccessibility because their stability during gastric digestion differed [52]. Likewise, when vitamin D_3_ and eicosapentaenoic acid (EPA)/DHA nanoemulsions were incorporated into yogurt and fruit-puree desserts, their bioaccessibility changed significantly despite using the same initial nanoemulsion formulations [53]. These findings show that food-matrix properties, including viscosity, protein–lipid interactions, matrix breakdown, and droplet release during digestion, can strongly influence nutrient delivery.

Overall, droplet size is an important structural parameter, but it should not be treated as an independent predictor of gastrointestinal delivery. The performance of ultrasound-assisted nanoemulsions depends on a sequence of linked events: droplet disruption, interfacial renewal, colloidal stability during digestion, lipid hydrolysis, mixed-micelle formation, and finally bioaccessibility. Future studies should compare compositionally identical formulations that differ only in droplet size, while measuring lipolysis, micellarization, and bioaccessibility together. Such designs are needed to clarify how post-sonication droplet size actually relates to gastrointestinal delivery.

### 4.3. Ultrasound-Assisted Delivery Platforms Beyond Conventional Nanoemulsions

The performance of ultrasound-assisted delivery systems depends not only on the physicochemical properties of the encapsulated bioactive but also on the structural characteristics of the carrier platform. Beyond conventional oil-in-water nanoemulsions, ultrasound has been successfully applied to protein-based nanocarriers and emulsion-gel systems, where acoustic cavitation modifies interfacial organization, carrier structure, and encapsulation performance. Consequently, improvements in delivery are often achieved through multiple mechanisms, including enhanced emulsification, protein conformational modification, strengthened carrier–bioactive interactions, and optimized interfacial architecture, rather than droplet-size reduction alone.

Lipid-based nanoemulsions remain the most extensively studied ultrasound-assisted delivery platform for lipophilic nutrients. Fish-oil-based vitamin D nanoemulsions prepared by ultrasonication exhibited high encapsulation efficiency (95.7–98.2%), excellent storage stability, and improved vitamin D bioaccessibility following simulated gastrointestinal digestion [45]. Similarly, ultrasound-assisted modification of octenyl succinic anhydride (OSA)-rice starch enhanced the encapsulation performance of curcumin delivery systems, increasing encapsulation efficiency from 81.65% to 89.03% by improving the functional properties of the carrier material rather than simply reducing particle size [54]. These studies demonstrate that ultrasound can simultaneously improve carrier functionality and digestive performance through interfacial engineering.

Protein-based nanocarriers provide an additional strategy for delivering poorly water-soluble phytochemicals. High-intensity ultrasound pretreatment of soy protein isolate (SPI) substantially improved quercetin loading, increasing encapsulation efficiency from 7.2% in native SPI to 76.5% after ultrasound treatment, while increasing in vitro bioaccessibility from 10.5–13.0% for free quercetin to 61.1–64.5% after nanocomplex formation [55]. These improvements were attributed to ultrasound-induced structural modification of SPI, which exposed hydrophobic binding sites and strengthened protein–polyphenol interactions, thereby enhancing carrier functionality and digestive release.

Ultrasound has also shown considerable promise in emulsion-gel delivery systems, where it modifies protein–polysaccharide networks that regulate the encapsulation and release of bioactive compounds. In fermentation-induced SPI emulsion gels, optimization of ultrasound treatment increased quercetin encapsulation efficiency from 81.25% to 90.04% and improved bioaccessibility from 28.90% to 42.58% [56]. Likewise, ultrasound-assisted emulsification of myofibrillar protein–carboxymethyl cellulose (MP–CMC) emulsion gels enhanced curcumin encapsulation efficiency from 86.75% to 97.67% while increasing bioaccessibility from 28.68% to 44.93% [57]. These findings indicate that ultrasound can improve delivery performance by restructuring gel networks and strengthening interfacial interactions in addition to producing finely dispersed emulsions.

Together, these studies show that ultrasound does more than simply break droplets into smaller sizes. Depending on the delivery system, acoustic cavitation can improve encapsulation by altering protein structure, strengthening carrier–bioactive interactions, promoting interfacial adsorption, or reorganizing protein–polysaccharide networks [55,56,57]. Therefore, higher encapsulation efficiency should be viewed as evidence of better carrier performance, not as direct proof of greater gastrointestinal absorption.

Even with these advances, ultrasound-assisted delivery still depends strongly on formulation composition, interfacial stability, lipid digestion, and mixed-micelle formation [8,9]. Future studies should compare compositionally identical formulations prepared with and without ultrasound, while carefully controlling acoustic energy density and measuring encapsulation efficiency, lipolysis, micellarization, and, where relevant, in vivo pharmacokinetics. Such standardized comparisons are needed to separate the specific role of acoustic cavitation from the effects of carrier composition and formulation design.

### 4.4. Essential-Oil Nanoemulsions: Formulation-Dependent Antimicrobial Performance

Ultrasound-assisted nanoemulsification is an effective way to improve the dispersion, stability, and antimicrobial activity of essential oils (EOs) in food systems. Through acoustic cavitation, ultrasound generates local shear, microstreaming, and interfacial disruption, helping convert EO droplets into finer dispersions with better water compatibility and greater contact between antimicrobial compounds and microbial cells [36,58]. However, antimicrobial activity does not depend on droplet size alone. It is shaped by EO composition and concentration, the emulsifier system, interfacial properties, the target microorganism, and the food matrix [58].

Several studies show the broad potential of ultrasound-assisted EO nanoemulsions. Orange-peel, cinnamon, and oregano EO nanoemulsions prepared by ultrasound have produced stable nanoscale dispersions with antibacterial or antifungal activity against different microorganisms [17,19,59]. For example, cinnamon EO nanoemulsions showed strong antifungal activity against *Penicillium pinophilum*, while oregano EO nanoemulsions showed microorganism-dependent antibacterial and antifungal effects [19,59]. Orange-peel EO nanoemulsions also showed stronger antibacterial activity as EO concentration increased, suggesting that antimicrobial performance is closely linked to the availability and release of active compounds, not simply to smaller droplet size [17].

Recent studies have also tested ultrasound-assisted EO nanoemulsions in real food systems (Table 1). An optimized nanoemulsion containing oregano, thyme, and lemongrass EOs produced droplets below 100 nm and inhibited *Escherichia coli*, *Staphylococcus aureus*, and *Salmonella Enteritidis* in refrigerated trout fillets, extending shelf life [20]. Similarly, ultrasound-assisted oregano EO nanoemulsions combined with ultrasound treatment showed a synergistic bactericidal effect against *Listeria monocytogenes* during blueberry storage. The treatment increased membrane permeability, promoted oxidative damage, altered cell morphology, and improved microbial safety without reducing sensory quality [21]. These findings support the use of ultrasound-assisted EO nanoemulsions as part of hurdle-based preservation strategies for fresh foods [60].

Ultrasound-assisted EO nanoemulsions also show strong potential for active packaging applications. For example, oregano EO nanoemulsions incorporated into hydroxypropyl methylcellulose films improved antibacterial and antioxidant properties, indicating that nanoemulsification can enhance the dispersion and functional delivery of hydrophobic bioactives in biodegradable packaging materials [18]. In this study, ultrasonication reduced oregano EO droplet size from the macroemulsion scale (1420.47 ± 111.0 nm) to the nanoscale range (221.3 ± 0.80 nm) and improved size uniformity, which contributed to stronger antibacterial activity against *Escherichia coli* and *Staphylococcus aureus* [18]. However, the overall performance of active packaging systems depends not only on droplet size, but also on EO composition, nanoemulsion stability, polymer properties, and bioactive release behavior.

Overall, current evidence shows that ultrasound-assisted EO nanoemulsions can make natural antimicrobials more practical by improving their dispersion, stability, and delivery in complex food systems. However, their antimicrobial performance remains highly formulation-dependent, and comparison across studies is difficult because EO composition, emulsifier type, microbial targets, and testing protocols often differ. Future research should use more standardized designs that include acoustic characterization, detailed droplet-size analysis, minimum inhibitory and bactericidal concentrations, time–kill kinetics, biofilm testing, and validation in representative food matrices. This would make it easier to determine how much acoustic cavitation itself contributes to antimicrobial efficacy.

### 4.5. The Interface Paradox: Balancing Oxidative Stability and Digestive Performance

A key challenge in designing ultrasound-assisted nanoemulsions is the interface paradox: the enlarged oil–water interface that improves gastrointestinal delivery can also make unsaturated lipids more vulnerable to oxidation. Acoustic cavitation produces fine droplets with a large interfacial area, which can support lipase adsorption, lipid hydrolysis, and mixed-micelle formation during digestion. At the same time, this larger interface exposes polyunsaturated fatty acids to oxygen, transition metals, and other pro-oxidants. As a result, oxidative stability depends not only on droplet size, but also on the composition and protective capacity of the interfacial layer [24]. Effective nanoemulsion design therefore requires a balance between digestive accessibility and oxidative protection.

Ultrasound may further influence oxidation because cavitation creates localized high-energy conditions that can promote oxygen transfer and radical formation. Ganguly et al. similarly reported that oxidative stability in ultrasound-assisted nanoemulsions depended mainly on the interaction among acoustic processing conditions, oil composition, and interfacial structure, with protein-based interfacial layers offering stronger protection than less protective emulsifier systems [28]. Controlled emulsion studies also show that smaller droplets can increase oxygen exposure and lipid hydroperoxide formation, although the final oxidation level still depends heavily on the interface and storage conditions [61].

Interfacial engineering offers a practical way to manage this stability–digestibility trade-off. For example, fish-oil/α-tocopherol nanostructured lipid carriers achieved nanoscale particles of about 119 nm while significantly improving oxidative stability by reducing peroxide value and thiobarbituric acid-reactive substances during storage [62]. Although these systems were not produced by ultrasonication, they demonstrate a principle directly relevant to ultrasound-assisted nanoemulsions: placing antioxidants in the right lipid phase and designing a protective interface can help protect oxidation-sensitive lipids without losing delivery efficiency.

Importantly, the same interfacial properties that influence oxidation also shape gastrointestinal digestion. Long-chain omega-3 algal-oil nanoemulsions prepared with different emulsifiers showed clear differences in colloidal stability and digestive behavior despite having similar particle sizes [63]. This confirms that emulsifier selection affects both oxidative stability and nutrient release. Therefore, optimization should not focus only on making droplets smaller or increasing bioaccessibility; without adequate interfacial protection, these gains may come at the cost of chemical stability.

## 5. Challenges and Future Perspectives for Industrial Translation

### 5.1. Ultrasound-Assisted Nanoemulsification in the Context of Other High-Energy Technologies

Ultrasound-assisted nanoemulsification is one of several high-energy methods used to produce food-grade nanoemulsions. Other widely used technologies include high-pressure homogenization (HPH), microfluidization (MF), and ultra-high-pressure homogenization (UHPH). Each method reduces droplet size through intense mechanical forces, but the best choice depends on the formulation, production scale, energy demand, product quality requirements, and final food application rather than on one method being universally superior [64,65,66,67].

Ultrasound is distinct because it works through acoustic cavitation, which generates localized shear, microjets, shock waves, and interfacial disruption. These effects can break droplets and renew the oil–water interface, often producing stable nanoemulsions with relatively simple equipment and short processing times. This makes ultrasound especially useful for thermolabile bioactive compounds and laboratory-scale formulation development [8,36]. By contrast, HPH and MF force emulsions through high-pressure valves or microchannels, producing uniform droplets with strong industrial scalability, although they usually require higher capital investment and operating pressure [65,66].

Comparative studies show that ultrasound, HPH, and MF can all produce high-quality nanoemulsions, but their performance is highly formulation-dependent. For example, ultrasound-produced gamma-oryzanol nanoemulsions achieved smaller droplets, around 30 nm, than corresponding HPH emulsions, around 107 nm, while maintaining good storage stability [68]. Ultrasound has also produced nanoemulsions with narrow size distributions and high encapsulation efficiency for several lipophilic bioactives [9,11,45]. However, such results should not be generalized across all systems because oil type, emulsifier choice, viscosity, and processing conditions strongly affect the outcome.

Technology selection should therefore go beyond droplet size. Ultrasound offers flexibility for small- to moderate-scale processing, rapid formulation screening, and heat-sensitive ingredients, whereas HPH, MF, and UHPH currently have stronger industrial maturity for large-scale continuous production [65,66]. Hybrid strategies that combine ultrasound with pressure-based homogenization or other stabilization approaches may be especially useful, as they can bring together the interfacial flexibility of cavitation with the robustness of established industrial processes [69].

Overall, ultrasound-assisted nanoemulsification should be viewed as a complementary technology within the wider food emulsification toolkit, not as a direct replacement for high-pressure methods. Future comparisons should use standardized formulations and shared performance measures, including energy consumption, encapsulation efficiency, storage stability, oxidative stability, gastrointestinal bioaccessibility, processing cost, and environmental impact. This would provide a stronger basis for selecting the most suitable emulsification method for specific food and nutraceutical applications.

### 5.2. Industrial Scale-Up and Process Optimization

Despite strong progress at the laboratory scale, industrial application remains a major challenge for ultrasound-assisted nanoemulsification. Nanoemulsions optimized in small batches cannot simply be scaled up by increasing processing volume or ultrasonic power, because acoustic cavitation is highly dependent on reactor geometry, acoustic-field distribution, residence time, flow behaviour, and temperature control [26,70]. Successful scale-up therefore requires maintaining effective cavitation and comparable energy delivery while increasing production throughput.

Continuous-flow ultrasonic systems offer one of the most practical routes for scale-up. Compared with batch processing, they provide better control over residence time, temperature, and exposure to the cavitation field, improving process reproducibility [23]. Current strategies increasingly focus on continuous operation, enlarged radiating surfaces, and reactor numbering-up rather than relying only on larger ultrasonic probes [23,26].

Evidence from Peshkovsky and Bystryak, who produced an MF59^®^-analog nanoemulsion across laboratory, bench, and industrial scales using continuous-flow ultrasonic processors, shows that comparable droplet characteristics can be maintained when acoustic conditions are carefully controlled [23]. Although this example comes from the pharmaceutical sector, it provides a useful model for food and nutraceutical applications.

Energy efficiency and process economics are equally important for commercial adoption. Ultrasound may reduce processing time and offer flexibility for selected formulations, but its energy demand depends on amplitude, treatment time, flow rate, reactor design, and product composition [14,70]. Reporting nominal power alone is therefore insufficient. Future studies should use standardized metrics such as specific energy input, acoustic energy density, droplet-size distribution, and processing throughput to allow meaningful comparison between laboratory, pilot, and industrial systems [14,26].

Another practical barrier is the complexity of real food matrices. Many laboratory studies use simplified oil–water systems, whereas commercial foods contain proteins, polysaccharides, sugars, minerals, and other ingredients that affect viscosity, acoustic attenuation, cavitation intensity, droplet disruption, and emulsion stability [8,65]. Therefore, scale-up studies should include representative food matrices at the pilot stage rather than relying only on model emulsions. The main industrial challenges and possible mitigation strategies are summarized in Table 2.

Overall, industrial translation will require coordinated optimization of formulation composition, acoustic conditions, reactor design, energy use, and product quality. Future work should move from proof-of-concept studies toward pilot-scale validation that evaluates processing efficiency, stability, cost, and environmental impact under realistic manufacturing conditions.

### 5.3. Safety Considerations, Regulatory Aspects, and Future Research Priorities

The commercialization of ultrasound-assisted nanoemulsions depends not only on technical performance but also on safety, regulatory acceptance, and manufacturing consistency. When approved food-grade ingredients and appropriate processing conditions are used, ultrasound does not inherently compromise product safety. However, excessive cavitation can generate localized high temperatures, pressures, and reactive species, which may accelerate lipid oxidation or degrade oxidation-sensitive bioactive compounds [24,28,70]. Processing conditions must therefore be optimized to achieve efficient emulsification while preserving nutritional and chemical stability.

From a regulatory standpoint, the safety of ultrasound-assisted nanoemulsions is mainly determined by the final product composition, including the oils, emulsifiers, antioxidants, and encapsulated bioactives used. These components must comply with existing food regulations, while the manufacturing process must ensure reproducibility, storage stability, and consistent product quality. Key characterization parameters should include droplet-size distribution, encapsulation efficiency, oxidative stability, storage stability, and, where relevant, standardized gastrointestinal bioaccessibility [8,24].

Several gaps still limit commercial relevance. Many studies remain restricted to laboratory-scale model systems, use different ultrasonic conditions, and report acoustic parameters inconsistently. In addition, most investigations focus on physicochemical properties or simulated digestion, while fewer evaluate long-term storage, sensory quality, consumer acceptance, in vivo bioavailability, or techno-economic feasibility [14,23,26,65,70,76].

Future research should therefore place greater emphasis on standardized reporting, representative food matrices, pilot-scale testing, and direct comparison with established technologies such as high-pressure homogenization and microfluidization.

Future development should also move beyond the single goal of reducing droplet size. More attention is needed on interfacial engineering, antioxidant localization, lipid digestibility, food-matrix interactions, and product functionality. Emerging approaches such as Pickering emulsions, multilayer interfaces, protein–polysaccharide complexes, and active packaging systems may help improve the stability, delivery performance, and shelf life of ultrasound-assisted nanoemulsions. Combining these strategies with techno-economic assessment, life-cycle analysis, and regulatory evaluation will be essential for translating this technology into practical food and nutraceutical products.

## 6. Conclusions and Future Perspectives

Ultrasound-assisted nanoemulsification is a promising non-thermal technology for producing food-grade nanoemulsions, mainly through acoustic cavitation. The mechanical effects of cavitation, including shear forces, microjets, shock waves, and microstreaming, can disrupt coarse droplets and generate fine emulsions. However, successful nanoemulsion formation depends not only on ultrasound intensity, but also on formulation composition, emulsifier type, oil phase, processing time, energy input, and reactor design. Excessive sonication may provide little additional droplet-size reduction and can instead increase heating, lipid oxidation, or degradation of sensitive bioactives. Therefore, ultrasound should be optimized, not simply intensified.

Current evidence shows that ultrasound-assisted nanoemulsions can improve the encapsulation, dispersion, stability, and in vitro bioaccessibility of lipophilic compounds such as curcumin, vitamin D, fucoxanthin, and essential oils. However, improved bioaccessibility should not be treated as direct proof of increased systemic bioavailability, which still requires stronger in vivo and human validation. Similarly, droplet size is an important structural parameter, but it is not an independent predictor of gastrointestinal performance. Interfacial composition, lipid digestibility, colloidal stability during digestion, and food-matrix interactions are equally important.

A central challenge is the interface paradox: the enlarged oil–water interface can improve lipid digestion and micellarization, but it can also increase oxidative susceptibility, especially in systems containing unsaturated lipids. This makes interfacial engineering critical. Appropriate emulsifier selection, antioxidant localization, multilayer interfaces, protein–polysaccharide complexes, and Pickering stabilization may help balance digestive accessibility with oxidative protection.

For industrial translation, ultrasound should be viewed as a complementary technology rather than a replacement for high-pressure homogenization or microfluidization. Continuous-flow processing, reactor numbering-up, better acoustic characterization, and pilot-scale validation in real food matrices are needed to improve scalability and reproducibility. Future research should prioritize standardized reporting of acoustic parameters, compositionally controlled comparisons between sonicated and non-sonicated systems, pharmacokinetic validation, techno-economic analysis, and environmental assessment. With stronger integration of food chemistry, colloid science, nutrition, process engineering, and manufacturing, ultrasound-assisted nanoemulsification can move from a promising laboratory method toward broader application in food and nutraceutical systems.

## Figures and Tables

**Figure 1:**
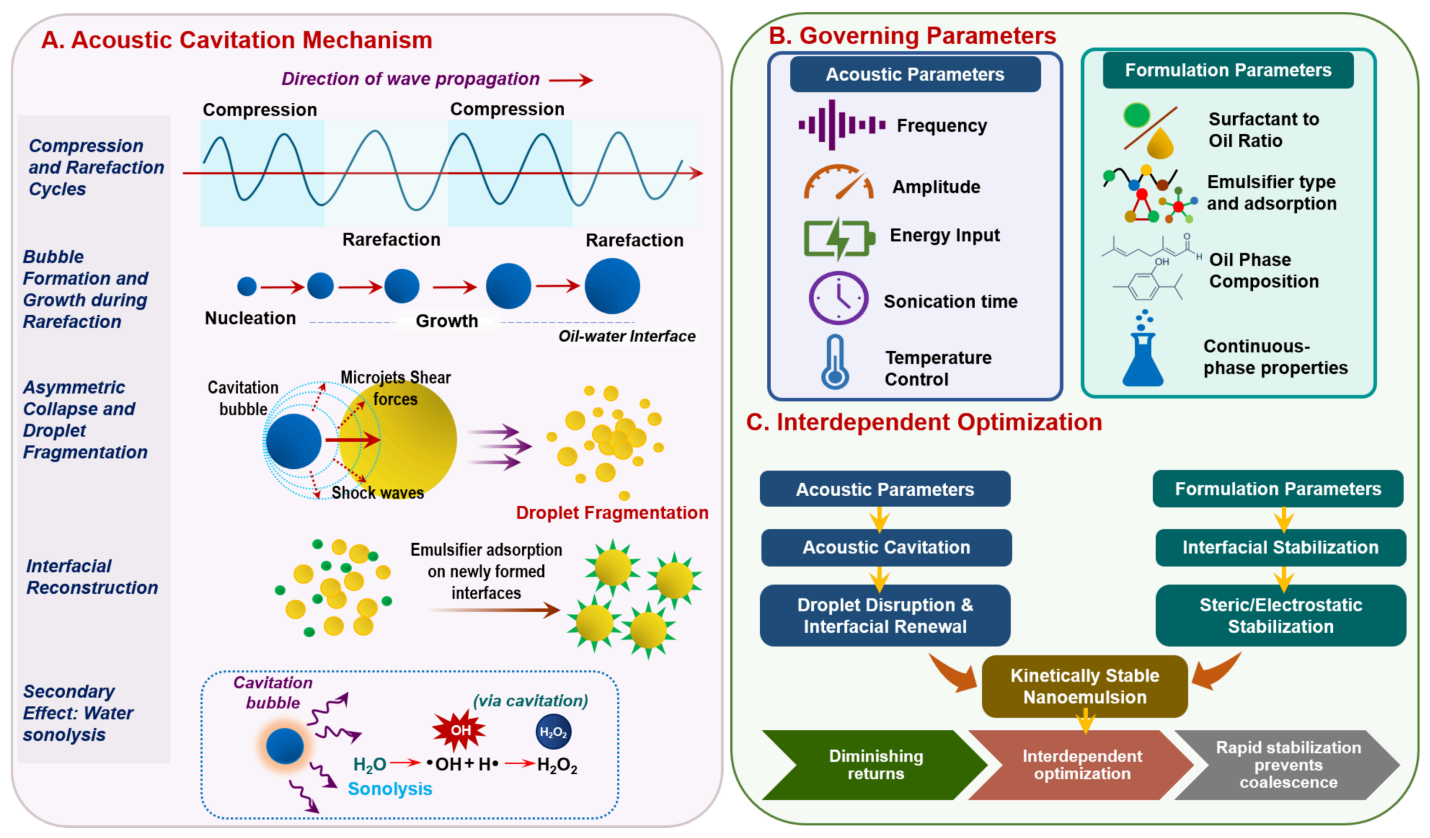
Schematic overview of ultrasound-assisted nanoemulsification. (**A**) Acoustic cavitation mechanism showing bubble formation, collapse, mechanical effects, and secondary ROS generation. (**B**) Governing acoustic and formulation parameters. (**C**) Interdependent optimization that leads to kinetically stable nanoemulsions, highlighting key insights on diminishing returns, collaborative enhancement, and rapid stabilization to prevent coalescence.

**Figure 2:**
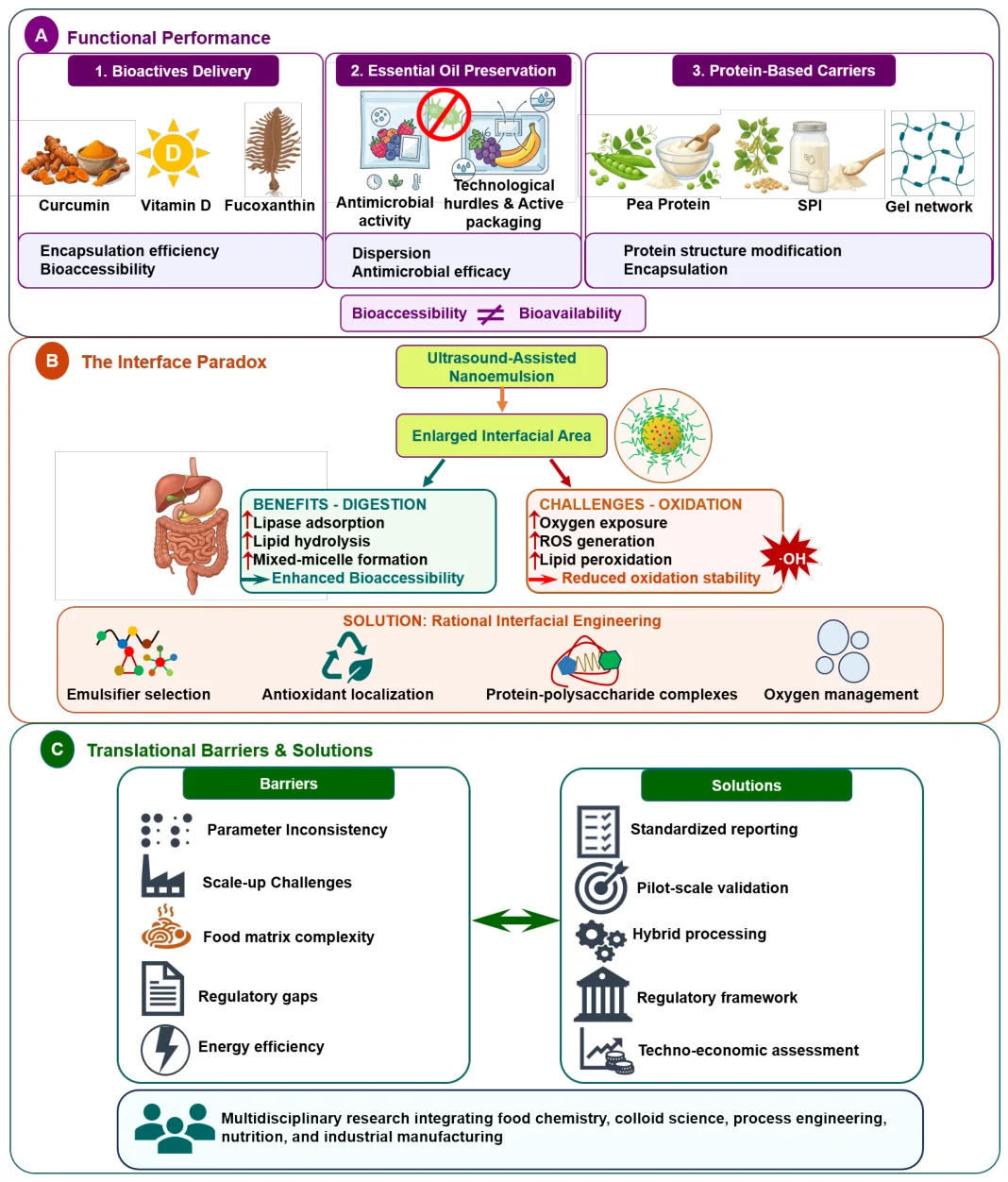
From laboratory to application: functional performance and translational barriers of ultrasound-assisted nanoemulsions. (**A**) Functional performance in bioactive delivery, essential oil preservation, and protein-based carriers. (**B**) The interface paradox: enlarged interfacial area promotes digestion but increases oxidative susceptibility, resolved by interfacial engineering. (**C**) Translational barriers and solutions for industrial adoption.

**Table 1:** Representative ultrasound-assisted essential-oil nanoemulsion formulations and their antimicrobial performance in food applications.

Nanoemulsion Formulation	Droplet Size Characteristic	Food Application/Antimicrobial Target	Main Antimicrobial Outcome	Formulation-Dependent Insight	Ref
Orange peel essential-oil nanoemulsion	9.70–26.60	*Listeria monocytogenes* and *Escherichia coli*	Strong antibacterial activity, with efficacy increasing as EO concentration increased	Antimicrobial performance depended on both essential-oil composition and nanoemulsion formulation rather than droplet size alone.	[17]
Cinnamon essential-oil nanoemulsion	<22	*Penicillium pinophilum*	Strong antifungal activity with stable nanoemulsion characteristics	Fine droplets produced by ultrasound-assisted processing improved nanoemulsion stability and antifungal performance.	[59]
Oregano EO, thymol and carvacrol nanoemulsions	54.47–84.07	*Staphylococcus aureus*, *Candida albicans* and *Aspergillus niger*	Broad-spectrum antibacterial and antifungal activity	Antimicrobial efficacy varied with the encapsulated bioactive compound, indicating that chemical composition was as important as droplet size.	[19]
Optimized antibacterial essential-oil blend nanoemulsion	<100 nm; PDI 0.17 ± 0.02	*E. coli*, *S. aureus* and *Salmonella Enteritidis* in rainbow trout fillets	Reduced bacterial growth and improved microbiological quality during refrigerated storage	Demonstrated successful translation of ultrasound-assisted EO nanoemulsification into a real food matrix.	[20]
Oregano essential-oil nanoemulsion combined with ultrasound	Z-average 132.43 ± 6.15 nm; PDI 0.26 ± 0.03; ζ-potential 26.14 ± 0.78 mV	*Listeria monocytogenes* and blueberry preservation	Synergistic bactericidal effect and improved microbial safety of blueberries	Nanoemulsion stability combined with ultrasound-induced cellular damage enhanced antimicrobial performance.	[60]
Oregano essential-oil nanoemulsion incorporated into HPMC composite films	221.3 ± 0.80 nm; PDI 0.251 ± 0.00 (macroemulsion: 1420.47 ± 111.0 nm; PDI 0.709 ± 0.04)	Active food packaging; *E. coli* and *S. aureus*	Greater antibacterial activity than macroemulsions after ultrasonication	Reduced droplet size and increased homogeneity improved EO dispersion, release, and antibacterial functionality in composite films.	[18]

**Table 2:** Major challenges and representative mitigation strategies for the industrial translation of ultrasound-assisted nanoemulsification.

Challenge	Scale-Up Implication	Evidence-Based Strategy	References
Reduced acoustic effectiveness at larger volumes	Lower or spatially uneven emulsification may increase droplet size and processing time	Use continuous-flow configurations, larger high-amplitude radiating surfaces, or ultrasound combined with mechanical stirring	[23,71]
Inadequate control of delivered energy	Nominal equipment power does not adequately describe emulsification performance across systems or scales	Measure and optimize acoustic power and energy density together with amplitude, treatment time and processing volume	[72,73]
Limitations of batch processing	Increasing batch volume reduces the proportion of product effectively exposed to the active acoustic zone	Apply continuous processing with controlled flow rate and residence time where higher throughput is required	[23,74]
Formulation-dependent emulsification	Emulsifier type and concentration, oil fraction and interfacial adsorption affect the energy required to obtain small, stable droplets	Optimize ultrasonic conditions using the intended food formulation rather than transferring conditions from simplified model systems	[71,74]
Thermal effects and excessive energy input	Heat accumulation and diminishing droplet-size reduction can reduce process efficiency and alter emulsion properties	Use temperature control and determine the minimum energy density required to achieve the target droplet size and stability	[72,73,75]

## Data Availability

No data was used for the research described in the article.
